# Biomarkers and the quadriceps femoris muscle architecture assessed by ultrasound in older adults with heart failure with preserved ejection fraction: a cross-sectional study

**DOI:** 10.1007/s40520-022-02189-7

**Published:** 2022-08-08

**Authors:** Iván José Fuentes-Abolafio, Michele Ricci, María Rosa Bernal-López, Ricardo Gómez-Huelgas, Antonio Ignacio Cuesta-Vargas, Luis Miguel Pérez-Belmonte

**Affiliations:** 1 Grupo de Investigación Clinimetría CTS-631, Departamento de Fisioterapia, Universidad de Málaga, C/Arquitecto Peñalosa, 3, 29071 Málaga, Spain; 2 Instituto de Investigación Biomédica de Málaga y Plataforma en Nanomedicina–IBIMA, Plataforma Bionand, Málaga, Spain; 3 Departamento de Medicina Interna, Hospital Regional Universitario de Málaga, Málaga, Spain; 4 CIBER Fisio-Patología de La Obesidad Y La Nutrición, Instituto de Salud Carlos III, Madrid, Spain; 5 School of Clinical Sciences, Faculty of Health at the Queensland University of Technology, Brisbane, QLD Australia; 6 Unidad de Neurofisiología Cognitiva, Centro de Investigaciones Médico Sanitarias (CIMES), Universidad de Málaga (UMA), Campus de Excelencia Internacional (CEI) Andalucía Tech, Málaga, Spain; 7 Centro de Investigación Biomédica en Red Enfermedades Cardiovasculares (CIBERCV), Instituto de Salud Carlos III, Madrid, Spain

**Keywords:** Blood biomarkers, Heart failure, Muscle thickness, Older adults, Ultrasound, Urinary biomarkers

## Abstract

**Background:**

Sarcopenia is an important comorbidity in patients with heart failure with preserved ejection fraction (HFpEF). The ultrasound (US) assessment has all the advantages of being used in primary care to assess muscle quantity and quality. Some biomarkers could be indicative of muscle mass loss.

**Aims:**

To describe the quantitative and qualitative characteristics of the quadriceps femoris assessed by US in older adults with HFpEF and to assess the relationship of the blood and urinary biomarkers, the polypharmacy and comorbidities with US outcomes in older adults with HFpEF.

**Methods:**

A cross-sectional study was conducted. 76 older adults with HFpEF were included. The quadriceps femoris muscle thickness (MT, cm), the subcutaneous fat tissue thickness (FT, cm), the muscle echo intensity (MEI) and the subcutaneous fat tissue echo intensity (FEI) were assessed by US in a non-contraction (non-con) and contraction (con) situations. Polypharmacy, comorbidities, blood and urine biomarkers were also collected.

**Results:**

The carbohydrate antigen 125 (CA-125), the folic acid and the urine creatinine shared the 86.6% variance in the non-con MT, adjusted by age, sex and body mass index (BMI). The folic acid shared the 38.5% of the variance in the con MT, adjusted by age, sex and BMI. The glycosylated haemoglobin explained the 39.6% variance in the non-con MEI, adjusted by age, sex and BMI. The chlorine (Cl^−^) explained the 40.2% of the variance in the non-con FT, adjusted by age, sex and BMI. The polypharmacy and the folic acid explained the 37.9% of variance in the non-con FEI, while the polypharmacy and the thyrotropin (TSH) shared the 44.4% of variance in the con FEI, both adjusted by age, sex and BMI. No comorbidities, polypharmacy, or blood and urinary biomarkers could explain the con MEI and the con FT variance.

**Conclusions:**

Blood and urinary biomarkers obtained in routine analyses could help clinicians detect US outcome changes in older adults with HFpEF and identify a worsening of sarcopenia.

**Trial registration:**

NCT03909919. April 10, 2019. Retrospectively registered.

**Supplementary Information:**

The online version contains supplementary material available at 10.1007/s40520-022-02189-7.

## Introduction

Heart Failure (HF) is a chronic and clinical syndrome with symptoms and/or signs caused by a structural or functional cardiac abnormality [1, 2]. Although the HF worldwide prevalence ranges from 1 to 3%, the number of patients with HF is increasing due to the population ageing [3]. It is estimated that 50% of patients with HF have a preserved ejection fraction (HFpEF) [3, 4]. Comorbidity is common in patients with HFwith reduced ejection fraction (HFrEF) but slightly more severe in HFpEF [5]. Approximately 50% of patients with HFpEF have five or more major comorbidities [5]. Sarcopenia has been acknowledged as an important comorbidity in patients with HF [6, 7]. The sarcopenia prevalence is about 19.7% in patients with HFpEF [8].

Many factors related to HF potentially lead to sarcopenia, like hormonal changes, physical inactivity, oxidative stress or inflammation [9, 10]. Sarcopenia could reduce the aerobic capacity and the quality of life of patients with HFpEF [8]. Moreover, sarcopenia has also been associated with a worse prognosis in patients with HFpEF [11]. The 2018 European Working Group on Sarcopenia in Older People (EWGSOP2) recommended using low muscle strength and low muscle quantity and quality to confirm the sarcopenia diagnosis [12]. The EWGSOP2 suggested that the Skeletal Muscle Mass (SMM) or Appendicular Skeletal Muscle Mass (ASM) delimit the muscle quantity [12]. Magnetic resonance imaging (MRI) and computed tomography (CT) are gold standards for the noninvasive assessment of muscle quantity. In contrast, Dual-energy X-ray absorptiometry (DXA) is considered the reference standard [13, 14]. However, these tools are not commonly used in primary care because of high equipment costs and the lack of portability [13, 14]. Ultrasound (US) assessment has shown validity in estimating muscle quantity compared to DXA, MRI and CT [15]. The US is a portable, cheap, simple, easy-to-use, and widely available clinical practice technique that can be performed bedside and enables the physician to visualize the muscle quantity and quality with high resolution within a relatively short period [16, 17]. Thus, the US has all the advantages to be used in clinical practice and has become the standard tool for sarcopenia screening [16, 17]. US assessment has also been a reliable and valid tool to assess the muscle quantity of pennate muscles, such as the quadriceps femoris, in older adults [15]. The quadriceps femoris imaging has shown to be a good predictor of whole-body skeletal muscle mass [18, 19]. The quadriceps femoris has also become the muscle most investigated because it is easy to measure and can be linked directly to measures of physical performance [20, 21]. In this context, Kawai et al. [22] reported the possibility of using the quadriceps femoris muscle’s US quantitative and qualitative characteristics to assess muscle strength, physical function, and sarcopenia in community-dwelling older adults.

Some blood and urinary biomarkers have also been associated with sarcopenia [23–30]. Thus, urine creatinine correlated well with MRI-based measures of muscle quantity and modestly with measures from bioelectrical impedance analysis (BIA) and DXA, resulting in a good proxy measure for estimating whole-body muscle mass [26, 31]. It is unlikely that a single biomarker can identify sarcopenia because of the complex pathophysiology, so the development of a panel of biomarkers must instead be considered [27, 32, 33]. It would be important that biomarkers could detect pathophysiological mechanisms related to sarcopenia before sarcopenia clinical manifestations (reduced aerobic fitness, exercise intolerance or reduced physical performance) appear in patients with HFpEF. Blood and urinary analyses are frequent in clinical practice to assess the evolution of HF and the patient's health status. It would be interesting for physicians to have biomarkers obtained from these routine analyses related to systemic dysfunction, which could aggravate the pathophysiological mechanisms that lead to loss of muscle quantity or quality [27]. Furthermore, these biomarkers could also monitor the effectiveness of a treatment on the pathophysiology of sarcopenia [27].

Thus, the objectives of the present study are (1) to assess the muscle thickness (MT), the subcutaneous fat tissue thickness (FT), the muscle echo intensity (MEI) and the subcutaneous fat tissue echo intensity (FEI) of the quadriceps femoris assessed by US in older adults with HFpEF and (2) to assess the relationship of the blood and urinary biomarkers, the polypharmacy and the comorbidities with these quantitative and qualitative characteristics of the quadriceps femoris in older adults with HFpEF.

## Methods

### Design and participants

A cross-sectional study was conducted. Seventy-six older adults with HFpEF were recruited as volunteers between April 2019 and March 2020 from the Heart Failure Unit of the Internal Medicine Service at the Regional University Hospital of Malaga (Spain). Older adults with HFpEF included in this study met the following inclusion criteria: (1) patients with HFpEF older than 70 years and diagnosed according to the European Society of Cardiology consensus statement [6]. Older adults with HFpEF were excluded if they met the following exclusion criteria: (1) older adults with HFpEF with a New York Heart Association (NYHA) class = 4; (2) older adults hospitalised ≤ 3 months; (3) older adults with a score on the Mini-Mental Status Examination (MMSE) < 24.

### Outcomes

#### US Outcomes

**Fig. 1:**
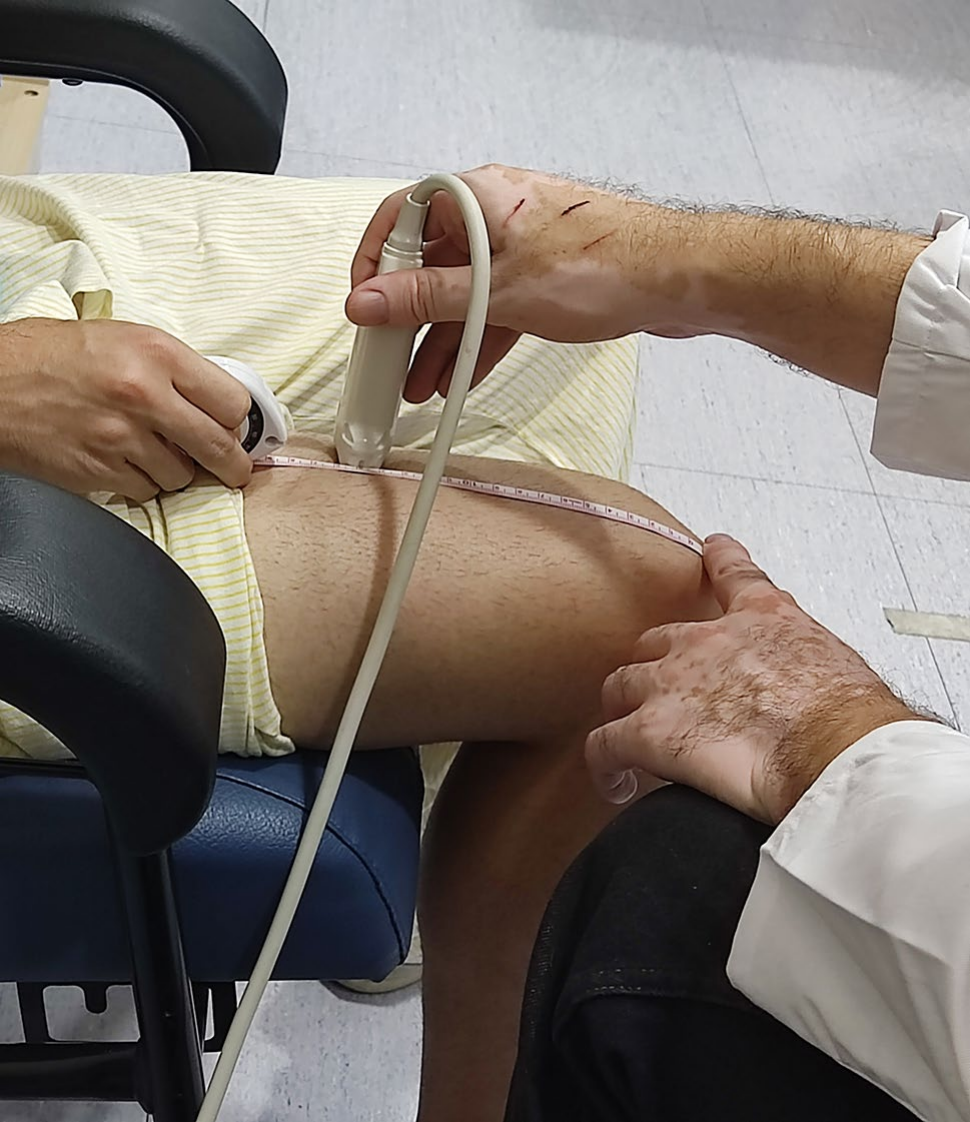
The landmark and the patients´ position for the ultrasound assessment

The right quadriceps femoris muscle and the subcutaneous fat tissue were measured in non-contraction (non-con) and contraction (con) situations. The US image was taken 15 cm from the upper edge of the patella, where transverse images were obtained with a B-mode US device (The Esaote MyLab One; Esaote, Genova, Italy) equipped with a linear array transducer 5 cm long. The transducer was placed transversely to the direction of the fibres and perpendicular to the axis of the limb. Patients were seated in a chair with their hip and knee at 90° of flexion (Fig. 1). In this same position, the patients performed a voluntary isometric contraction manually resisted by the clinician to assess the US outcomes in a con situation. Before performing the US measurements, the patients rested for 5 min to avoid bias in measuring MT and MEI muscle thickness and echo intensity [34]. The parameters used to acquire US images included the B-mode, a frequency of 10 MHz, 4 cm deep and 42% of the gain. Coupling gel was abundantly applied to minimise distortion generated by underlying tissues. Shaving was not needed. The following variables were analysed:

- *MT:* the quadriceps femoris (rectus femoris and vastus intermedius) MT was calculated using a perpendicular line to the horizontal axis from the midpoint of the femur to standardise the measurement. This line was placed between the femur and the superior fascia. The value was expressed in cm. This measurement showed a high intra-rater and inter-rater reliability, with an intraclass correlation coefficient (ICC) of 0.98 and 0.96, respectively [35]. This measurement also showed an absolute error between days of 0.017 cm [36].
- *FT:* the perpendicular line between the superior fascia and the skin. The value was also expressed in cm.
- *MEI:* the quadriceps femoris echo intensity was calculated from the selected range of interest. A histogram of the 8-bit grayscale is generated from the selected range of interest. This histogram analysed all the image pixels from 0, black, to 255, white. The average result of the resultant histogram means the MEI; that is, the MEI represents the mean pixel intensity. This outcome has no unit of measurement.
- *FEI:* the FEI was calculated the same way as the MEI. This outcome has no unit of measurement.

The combination of these variables (thickness and echo intensity) in different situations (non-con and con) and tissues (quadriceps femoris muscle and subcutaneous fat tissue) allowed obtaining eight variables: non-con MT, non-con MEI, con MT, con MEI, non-con FT, non-con FEI, con FT and con FEI.

#### Blood and urinary biomarkers

Blood and urine analyses performed prior to medical check-up visits collected the blood and urinary biomarkers. These analyses were performed in the hospital’s clinical laboratory. We choose blood and urine parameters which are routinely checked and do not need special analysis.

#### Secondary outcomes

*Clinical-epidemiological:* age, gender, educational level, marital status, number of comorbidities, medications, left ventricular ejection fraction (LVEF), NYHA class, number of falls in the last year, and smoking status.

*Anthropometric data*: weight, height, and body mass index (BMI).

### Ethical issues

The study was registered on the ClinicalTrial.gov database as NCT03909919. Ethical approval was obtained from the Provincial Ethics Committee of Malaga, Spain (26,032,020). The study was carried out following the Helsinki Declaration [37] and was implemented and reported according to the Strengthening the Reporting of Observational Studies in Epidemiology (STROBE) Statement [38, 39]. The STROBE checklist for this study is shown in supplementary appendix A. All participants in this study signed an informed consent form prior to enrolment.

### Sample size

The sample size was calculated using the software G Power 3.1.9.2 (University of Düsseldorf, Germany) and based on the following alternative hypothesis: the correlation magnitude that is going to be detected between the blood and urinary biomarkers and US outcomes will be 0.6 [31] with a significance level of 0.05 (error *α* < 5%), and statistical power of 0.8 (80%), a sample consisting of 76 older adults with HFpEF will be needed. The author LMP-B carried out the recruitment in his consultations and made it possible to obtain the estimated sample size.

### Data analysis

**Fig. 2:**
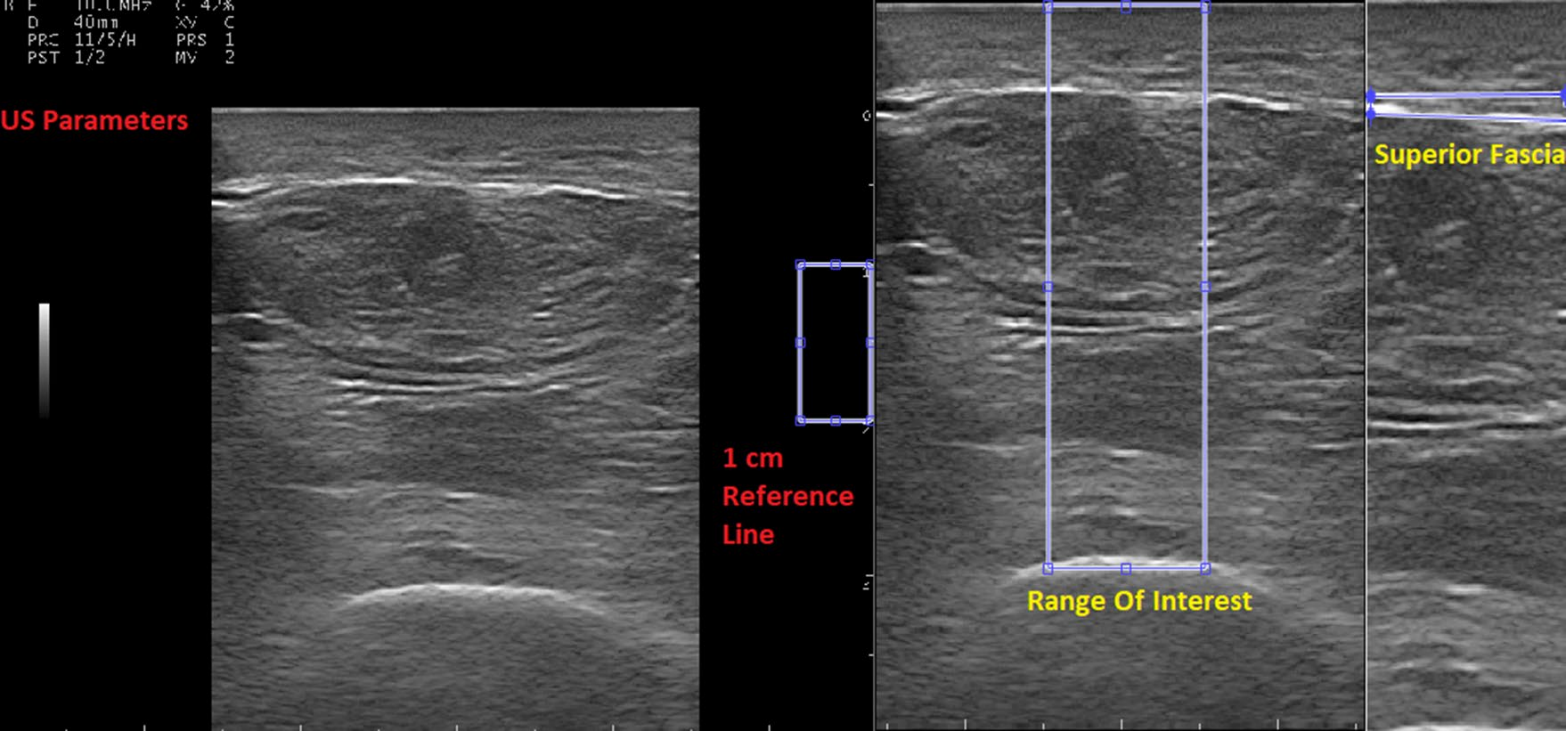
Range of interest and selected areas

US images were exported to a bmp file with 880 × 688 pixels resolution. MATLAB software (Version R2018b, MathWorks, Natick, USA) was used to process and analyse the US images. An own MATLAB code was created specifically for this project. This code allows the researcher to select a range of interest with a width of 1 cm and a height from the femur to the superficial layer of the skin (Fig. 2). Previously, the researcher had to record a reference line of 1 cm, relying on the line that shows the cm of the depth of the US image. That 1 cm reference line formed the width of the range of interest. Once the range of interest is selected, the code converts the image to grayscale. This type of assessment showed a high test–retest reliability score (ICC = 0.963) with an average coefficient of variation of 4.2% [40]. Then, the operator manually also selects the superior fascia to let the code distinguish between the muscular tissue (area between the femur and the superior fascia) and the subcutaneous fat tissue (area between the superior fascia and the skin) (Fig. 2). The code analyses the US outcomes using the selected areas. The same evaluator performed the MATLAB analyses of all images to reduce inter-rater variability.

Descriptive analyses and inferential analyses were carried out. An absolute frequency and a percentage are described as qualitative measures. Quantitative measures were reported using the mean and the standard deviation (SD) and through the maximum and the minimum. Distribution and normality were determined by one-sample Kolmogorov–Smirnov tests (significance < 0.05). When data from blood or urinary biomarker were missing, data imputation was performed in the Statistical Package for the Social Sciences (SPSS) using a multiple imputation analysis. The Pearson Correlation Coefficient (*r*) was used to assess the possible bivariate correlations between the US outcomes, blood and urinary biomarkers, comorbidities, and polypharmacy. Bivariate correlations were classified into three categories: poor (*r* ≤ 0.49), moderate (0.50 ≤ *r* ≤ 0.74) and strong (*r* ≥ 0.75). The relationship of the urinary and blood biomarkers, comorbidities, and polypharmacy with the US outcomes was evaluated using multivariate linear regression analysis. Two multivariate linear regression analyses were performed for each US outcome. The first analysis consisted of those variables that showed a significant bivariate correlation (*p* < 0.05) with the selected US outcome. The second analysis was formed with those variables that were significant in the first multivariate linear regression analysis, adjusted by age, sex and BMI. This second multivariate linear regression analysis was the model shown in the present study. The contribution of the exposures to the model’s predictability was assessed by the coefficient of determination (*R*^2^). A *p* value of *p* < 0.05 was considered to be statistically significant. All statistical analyses were conducted using the SPSS 22.0 for Windows.

## Results

### Patients’ characteristics

Data from the 76 included older adults with HFpEF were analysed without data loss from any patient. The acid folic, carbohydrate antigen 125 (CA-125), the urine creatinine and the aspartate transaminase (AST) needed the imputation of some data to reach the sample of *n* = 76. The descriptive, anthropometric and clinical variables are shown in Supplementary Appendix B. Descriptive statistics of the study outcomes are reported in Table 1. The mean age of older adults with HFpEF was 80.75 years old, and the mean LVEF was 60.74. Forty-five older adults with HFpEF (59.20%) were women, and 53 older adults showed an NYHA = 2 (69.70%). Most of the included older adults with HFpEF showed an overweight (40.8%), and 42 older adults (55.30%) had fallen in the past 12 months. Moreover, included older adults showed an average of 8.41 comorbidities and took 10.18 drugs every day. The most frequent comorbidities were arterial hypertension (97.40%), dyslipidemia (86.80%), heart valve disease (65.80%), and chronic renal insufficiency (64.50%). Furthermore, 72 older adults with HFpEF (94.70%) showed a left ventricular dilatation, while 41 older adults with HFpEF (53.90%) showed left ventricular hypertrophy, and 37 of these older adults (48.70%) reported a left atrial dilatation. The drugs that were most taken were loop diuretics (85.50%), beta-blockers (73.70%) and the angiotensin II receptor blocker (61.80%). Older adults with HFpEF showed a mean non-con MT of 2.06 cm (0.53) while the con MT was 2.40 cm (0.56). The non-con FT (1.12 cm) was larger than the con FT (1.08 cm). The non-con MEI (128.47) and the con MEI (125.61) were also larger than the non-con FEI (63.15) and the con FEI (52.22), respectively.

**Table 1:** Descriptive statistics of the study outcomes (*n* = 76)

	Mean (SD)	Min–max
US Outcomes		
Thickness		
Non-con MT (cm)	2.06 (0.53)	0.97–3.57
Con MT (cm)	2.40 (0.56)	1.14–3.74
Non-con FT (cm)	1.12 (0.75)	0.23–3.49
Con- FT (cm)	1.08 (0.71)	0.23–3.28
Echo intensity		
Non-con MEI	128.47 (50.61)	45.48–254.64
Con MEI	125.61 (45.48)	46.75–238.87
Non-con FEI	63.15 (26.13)	16.87–137.68
Con FEI	52.22 (23.54)	8.85–124.92
Blood and urinary biomarkers		
Blood biomarkers		
Hb (g/dL)	13.93 (11.11)	6.40–108.0
MCV (fL)	95.51 (19.03)	72–247
Leukocytes count (× 10^9^/L)	8.26 (4.81)	3.54–45.20
Blood platelets (× 10^9^/L)	221.30 (70.98)	8–415
Glucose (mg/dL)	102.28 (28.42)	61–195
Creatinine (mg/dL)	1.35 (0.82)	0.51–6.33
GF (mL/min/1,73 m^2^)	50.88 (20.42)	8–90
Na^+^ (mEq/L)	139.76 (3.38)	124–148
K^+^ (mEq/L)	4.64 (0.49)	3.5–6.2
Cl^−^ (mEq/L)	101.41 (4.14)	89–111
Glycosylated haemoglobin (%)	6.37 (1.11)	4.8–10.4
Ferritin (ng/mL)	123.44 (140.65)	10–738
Transferrin saturation (%)	21.09 (25.26)	5–214
Total cholesterol (mg/dL)	160.76 (41.98)	98–317
LDL (mg/dL)	86.32 (29.76)	36–167
HDL (mg/dL)	46.78 (14.11)	28–90
NT-proBNP (pg/mL)	2230.38 (3485.57)	42–19,118
Vitamin D (ng/mL)	23.34 (10.57)	7–50
Vitamin B12 (pg/mL)	490.58 (642.82)	103–4668
ALT (U/L)	22.15 (13.38)	8–94
AST (U/L)	43.82 (7.02)	17–76
TSH (µIU/mL)	2.38 (1.81)	0.1–12.0
Albumin (g/dL)	3.97 (3.85)	2.30–36.00
Bilirubin (mg/dL)	0.61 (0.31)	0.1–1.8
CA-125 (U/mL)	52.71 (83.26)	2–525
Folic acid (ng/mL)	8.70 (5.63)	1–38
Urinary biomarkers		
Urine creatinine (mg/dL)	81.42 (49.13)	13.66–348.54

*SD* standard deviation, *US* ultrasound, *Non-con MT* non-contraction muscle thickness, *Con MT* contraction muscle thickness, *Non-con FT* non-contraction subcutaneous fat tissue thickness, *Con FT* contraction subcutaneous fat tissue thickness, *Non-con MEI* non-contraction muscle echo intensity, *Con MEI* contraction muscle echo intensity, *Non-con FEI* non-contraction subcutaneous fat tissue echo intensity, *Con FEI* contraction subcutaneous fat tissue echo intensity, *Hb* haemoglobin, *MCV* mean corpuscular volume, *GF* glomerular filtration, *Na*^*+*^ sodium, *K*^*+*^ potassium, *Cl*^*−*^ chlorine, *LDL* low density lipoproteins cholesterol, *HDL* high density lipoproteins cholesterol, *NT-proBNP* pro-brain natriuretic peptide, *ALT* alanine aminotransferase, *AST* aspartate transaminase, *TSH* thyrotropin, *CA-125* carbohydrate antigen 125

The bivariate correlations between the US outcomes, the muscle strength, blood biomarkers, urinary biomarkers, comorbidities, and polypharmacy are shown in Table 2. In summary, the non-con MT showed a significant but poor correlation with the polypharmacy (*r* = 0.255, *p* = 0.026), the urine creatinine (*r* = 0.256, *p* = 0.030) and the CA-125 (*r* = − 0.262, *p* = 0.031). The non-con MT also showed a significant and moderate correlation with the folic acid (*r* = 0.557, *p* < 0.001). The con MT showed a significant and moderate correlation with the folic acid (*r* = 0.532, *p* < 0.001) and a significant but poor correlation with thyrotropin (TSH) (*r* = − 0.247, *p* = 0.037). The non-con MEI a significant but poor correlation with the glomerular filtration (GF) (*r* = 0.245, *p* = 0.046) and the glycosylated haemoglobin (*r* = 0.360, *p* = 0.005), while the con MEI did not show any correlation. The non-con FT showed a significant but poor correlation with the chlorine (Cl^−^) (*r* = 0.230, *p* = 0.047). The con FT also showed a significant but poor correlation with the Cl^−^ (*r* = 0.249, *p* = 0.032) and the polypharmacy (*r* = 0.250, *p* = 0.029). The non-con FEI showed a significant but poor correlation with comorbidities (*r* = − 0.235, *p* = 0.041), the polypharmacy (*r* = − 0.447, *p* < 0.01), the Cl^−^ (*r* = 0.236, *p* = 0.041), the folic acid (*r* = − 0.294, *p* = 0.010) and the TSH (*r* = 0.246, *p* = 0.037). The con FEI showed a significant but poor correlation with the polypharmacy (*r* = − 0.429, *p* < 0.01), the Cl^−^ (*r* = 0.280, *p* = 0.015) and the TSH (*r* = 0.348, *p* = 0.003).

**Table 2:** Bivariate correlations (*r*) between the QFMT, the muscle strength, blood biomarkers, urinary biomarkers, comorbidities, and polypharmacy

	Comorbidities	Polypharmacy	Hb	MVC	Leukocytes Count	Blood Platelets	Glucose	Creatinine	NT-proBNP
Non-con MT	0.134	0.255*	− 0.033	0.028	− 0.018	0.011	− 0.037	-0.065	-0.076
Con MT	0.167	0.169	− 0.019	0.000	− 0.013	0.022	− 0.026	− 0.123	− 0.053
Non-con MEI	0.007	0.039	− 0.118	0.007	− 0.011	− 0.092	0.210	0.066	0.146
Con MEI	− 0.008	0.064	− 0.104	− 0.024	0.007	− 0.118	0.131	0.123	0.215
Non-Con FT	− 0.070	− 0.217	0.192	0.031	− 0.095	0.101	− 0.110	− 0.145	− 0.103
Con FT	− 0.052	− 0.250*	0.196	0.097	− 0.099	0.107	− 0.090	− 0.149	− 0.116
Non-con FEI	− 0.235*	− 0.447**	0.118	0.048	− 0.112	0.061	− 0.066	-0.118	− 0.014
Con FEI	− 0.177	− 0.429**	0.163	0.131	− 0.112	0.018	− 0.117	− 0.085	0.012

	GF	Na^+^	K^+^	Cl^−^	Ferritin	TSI	Glycosylated Haemoglobin	Cholesterol	LDL	HDL
Non-con MT	0.124	0.009	− 0.011	− 0.111	0.015	− 0.195	0.084	− 0.058	− 0.059	− 0.063
Con MT	0.175	0.026	− 0.140	− 0.074	− 0.032	− 0.210	0.087	− 0.049	− 0.057	− 0.096
Non-con MEI	0.245*	0.062	− 0.009	− 0.120	− 0.048	− 0.117	0.360**	0.103	0.035	− 0.078
Con MEI	0.162	0.026	0.050	− 0.060	0.056	− 0.053	0.251	0.061	0.050	− 0.163
Non-con FT	− 0.113	0.135	− 0.017	0.230^*^	− 0.024	0.020	− 0.190	− 0.045	− 0.017	0.153
Con FT	− 0.125	0.121	0.008	0.249^*^	− 0.049	0.018	− 0.195	− 0.040	− 0.019	0.197
Non-con FEI	− 0.033	0.199	− 0.050	0.236^*^	− 0.061	0.136	− 0.167	0.187	0.185	0.212
Con FEI	− 0.091	0.148	0.047	0.280^*^	0.043	0.211	− 0.209	0.088	0.130	0.192

	Vitamin D	Vitamin B12	ALT	CA-125	Urine Creatinine	AST	Albumin	Bilirubin	Folic Acid	TSH
Non-con MT	− 0.021	− 0.051	− 0.093	− 0.262*	0.256*	− 0.029	0.135	0.111	0.557**	− 0.171
Con MT	− 0.064	− 0.041	− 0.019	− 0.191	0.208	− 0.111	0.132	0.121	0.532**	− 0.247*
Non-con MEI	− 0.058	− 0.067	0.192	0.041	− 0.058	− 0.010	0.117	− 0.135	− 0.007	− 0.199
Con MEI	− 0.045	− 0.011	0.107	0.099	− 0.036	0.172	0.192	− 0.207	0.006	− 0.080
Non-con FT	0.060	− 0.006	− 0.173	− 0.082	0.089	− 0.075	− 0.122	0.199	0.090	0.183
Con FT	0.043	− 0.016	− 0.177	− 0.083	0.072	− 0.066	− 0.133	0.193	0.076	0.193
Non-con FEI	0.020	0.011	− 0.120	0.147	− 0.073	− 0.049	− 0.182	0.013	− 0.294*	0.246*
Con FEI	0.031	0.008	− 0.214	0.169	0.012	0.166	− 0.158	− 0.013	− 0.187	0.348**

*Non-con MT* non-contraction muscle thickness, *Con MT* contraction muscle thickness, *Non-con FT* non-contraction subcutaneous fat tissue thickness, *Con FT* contraction subcutaneous fat tissue thickness, *Non-con MEI* non-contraction muscle echo intensity, *Con MEI* contraction muscle echo intensity, *Non-con FEI* non-contraction subcutaneous fat tissue echo intensity, *Con MEI* contraction subcutaneous fat tissue echo intensity, *Hb* haemoglobin, *MCV* mean corpuscular volume, *GF* glomerular filtration, *Na*^*+*^ sodium, *K*^*+*^ potassium, *Cl*^−^ chlorine, *TSI* transferrin saturation index, *LDL* low density lipoproteins cholesterol, *HDL* high density lipoproteins cholesterol, *NT-proBNP* pro-brain natriuretic peptide, *ALT* alanine aminotransferase, *AST* aspartate transaminase, TSH thyrotropin, *CA-125* carbohydrate antigen 125

*p < 0.05 **p < 0.01

The summary of the multivariate linear regression models is shown in Table 3. Multivariate linear regression models are shown in Table 4. The CA-125, the folic acid and the urine creatinine shared 86.6% of the variance in the non-con MT, adjusted by age, sex and BMI. The folic acid shared 38.5% of the variance in the con MT, adjusted by age, sex and BMI. The glycosylated haemoglobin explained 39.6% variance in the non-con MEI, adjusted by age, sex and BMI. The Cl- explained 40.2% variance in the non-con FT, adjusted by age, sex and BMI. The polypharmacy and the folic acid explained 37.9% variance in the non-con FEI, while the polypharmacy and the TSH shared 44.4% of the variance in the con FEI, both adjusted by age, sex and BMI. No comorbidities, polypharmacy, or blood and urinary biomarkers could explain the con MEI and the con FT variance.

**Table 3:** Summary of Models

	*R*	*R*^2^	Adjusted *R*^2^	SE	*F*	*p*
Non-con MT	0.931	0.866	0.852	0.20	61.44	< 0.001
Con MT	0.621	0.385	0.350	0.45	11.12	< 0.001
Non-con MEI	0.629	0.396	0.351	40.15	8.85	< 0.001
Non-con FT	0.634	0.402	0.368	0.60	11.77	< 0.001
Non-con FEI	0.616	0.379	0.335	21.31	8.56	< 0.001
Con FEI	0.666	0.444	0.402	17.08	10.55	< 0.001

**Table 4:** Multivariate linear regression models

Dependent outcome	Predictor Variables	Non-standardized coefficients		Typified coefficients	*t*	*p*	95% CI
		B	SE	Beta			
Non-con MT	(Constant)	1.477	0.466		3.171	0.002	(0.544, 2.410)
	Ca-125	− 0.004	0.001	− 0.420	− 7.067	0.000	(− 0.005, − 0.003)
	Folic acid	0.175	0.011	1.971	15.947	0.000	(0.153, 0.197)
	Urine creatinine	− 0.016	0.001	− 1.520	− 13.385	0.000	(− 0.018, − 0.013)
	Sex	− 0.103	0.052	− 0.100	− 1.989	0.051	− 0.207, 0.001)
	Age	0.002	0.005	0.027	0.474	0.637	(− 0.008, 0.012)
	BMI	0.012	0.006	0.123	2.113	0.039	(0.001, 0.024)
Con MT	(Constant)	2.483	0.920		2.699	0.009	(0.649, 4.317
	Folic acid	0.044	0.009	0.470	4.958	0.000	(0.027, 0.062
	Sex	− 0.225	0.109	− 0.197	− 2.059	0.043	(− 0.443, − 0.007
	Age	− 0.011	0.010	− 0.118	− 1.167	0.247	(-0.031, 0.008
	BMI	0.020	0.010	0.209	2.046	0.044	(0.001, 0.040)
Non-con MEI	(Constant)	273.495	103.271		2.648	0.011	(66.450, 480.541)
	Glycosylated Haemoglobin	8.860	4.916	0.201	1.802	0.077	(− 0.995, 18.715)
	Sex	− 37.003	11.442	− 0.368	− 3.234	0.002	(− 59.943, − 14.064)
	Age	− 1.202	1.055	− 0.130	− 1.139	0.260	(− 3.318, 0.913)
	BMI	− 2.760	0.924	− 0.344	− 2.987	0.004	(− 4.612, − 0.907)
Non-con FT	(Constant)	− 6.090	1.971		− 3.090	0.003	(− 10.021, − 2.159)
	Cl^−^	0.044	0.017	0.245	2.601	0.011	(0.010, 0.078)
	Sex	0.429	0.143	0.284	2.992	0.004	(0.143, 0.715)
	Age	0.008	0.013	0.060	0.592	0.556	(− 0.018, 0.033)
	BMI	0.062	0.013	0.488	4.857	0.000	(0.037, 0.088)
Non-con FEI	(Constant)	36.089	48.014		0.752	0.455	(− 59.672, 131.850)
	Polypharmacy	− 3.418	0.824	− 0.412	− 4.150	0.000	(− 5.061, − 1.775)
	Folic acid	− 1.150	0.420	− 0.263	− 2.739	0.008	(− 1.987, − 0.313)
	Sex	12.627	5.150	0.239	2.452	0.017	(2.357, 22.898)
	Age	0.466	0.475	0.105	0.981	0.330	(− 0.481, 1.414)
	BMI	0.883	0.464	0.198	1.903	0.061	(− 0.042, 1.809)
Con FEI	(Constant)	60.051	39.544		1.519	0.134	(− 18.902, 139.004)
	Polypharmacy	− 2.808	0.674	− 0.399	− 4.166	0.000	(− 4.153, − 1.462)
	TSH	3.679	1.209	0.302	3.043	0.003	(1.265, 6.093)
	Sex	18.035	4.257	0.403	4.237	0.000	(9.536, 26.534)
	Age	0.064	0.393	0.017	0.163	0.871	(− 0.721, 0.849)
	BMI	− 0.174	0.413	− 0.046	− 0.422	0.674	(− 0.999, 0.650)

## Discussion

The present study showed the non-con MT, the con MT, non-con FT, con FT, non-con MEI, con MEI, non-con FEI and con FEI of the quadriceps femoris muscle. This study is the first study to report these US outcomes in older adults with HFpEF, apart from non-con MT, which has been reported previously. Our results reported that older adults with HFpEF have a mean non-con MT of 2.06 cm (0.53). Morimoto et al. [41] reported a median quadriceps femoris MT of 2.11 cm in patients with HFpEF and HErEF, which is similar to the results obtained by our study. Nakano et al.[42] showed a mean vastus lateralis, vastus medialis, rectus femoris and vastus intermedius MT of 5.21 cm (1.10) in patients with HFpEF and HFrEF. Nakano et al. [42] also showed that patients with HF have a reduced vastus lateralis, vastus medialis, rectus femoris, and vastus intermedius MT compared with healthy people (5.21 vs 6.54 cm). Sarcopenia is more prevalent in patients with HFpEF and HFrEF than in healthy older adults, so it seems logical that HF patients have lower MT than healthy older adults [43]. Sarcopenia has been acknowledged as an important comorbidity in patients with HF [6, 7]. Thus, sarcopenia could reduce the aerobic capacity, the quality of life and the exercise intolerance of patients with HFpEF [8, 44]. Moreover, sarcopenia has also been associated with a worse prognosis in patients with HFpEF [11]. Muscle strength and physical functional performance are easily measurable in primary care settings [13]. On the other hand, the US has all the advantages of being used in primary care to assess muscle quantity and quality [15–17]. Therefore, it is important to collect the US outcomes assessed in our study in different age cohorts and populations, such as older adults with HF [17]. This way, pathological values can be distilled, cut-off points can be established, and correlations with the other aspects of sarcopenia-strength and function [17]. Many different mechanisms related to HF potentially lead to sarcopenia, such as hyper-activation of the sympathetic system, systemic inflammation, elevated oxidative processes, higher apoptotic activity or reduced release of the skeletal muscle [9, 10, 43]. On the other hand, sarcopenia induces impaired muscle contraction and metabolic and endocrine abnormalities that may contribute to cardiovascular remodelling and dysfunction and the development of HFpEF [45]. Previous literature has also found accentuated muscle dysfunction, with reduced mitochondrial size in skeletal muscle and increased atrophy genes and protein levels, in stable outpatients with HFpEF compared with older adults with HFrEF and healthy controls [46]. These results show the importance of muscle wasting in older adults with HFpEF, which the US could assess.

The non-con MT showed a poor correlation with the polypharmacy (*r* = 0.255, *p* = 0.026). The Berlin Aging Study II showed a negative relationship between appendicular lean mass and polypharmacy [47]. However, the relationship between sarcopenia and polypharmacy seems unclear because it might vary according to patients’ heterogeneous health, age, functionality, nutritional characteristics, and comorbidities [48]. The non-con MT showed a poor bivariate correlation with the CA-125 (*r* = − 0.262, *p* = 0.031) and a moderate correlation with the folic acid (*r* = 0.557, *p* =  < 0.001). Our study is the first to report a relationship between non-con MT and CA-125 or folic acid.

It has been reported that there are higher levels of CA-125 in women with HFpEF than in healthy people [49]. A correlation between the CA-125 and the severity of systolic HF and the maximum left atrial volume has also been shown [49]. The CA-125 even could predict hospitalisation in women with HFpEF [49]. There is solid evidence of the usefulness of CA-125 as a biomarker for HF since it is easy to measure in a short period, uses widely available standardised methods, and is reasonably costly [50]. In addition, it is related to key pathophysiological processes in HF, such as fluid overload and inflammatory activity [50]. The inflammatory activity contributes to the loss of muscle mass [51]. The CA-125 is also associated with the severity of the disease and a worse prognosis [50]. It is useful for therapeutic follow-up and could be useful to guide treatment [50]. This study shows that CA-125 is also related to loss of muscle quantity in patients with HFpEF.

Decreases in folic acid intake may also impair muscle function through their action on homocysteine. In contrast, the folic acid supplementation plus a resistance training programme could significantly increase the muscle mass in older adults [52, 53]. The non-con MT also showed a poor and directly proportional correlation with the urine creatinine (*r* = 0.256, *p* = 0.030). Previous studies had reported a good correlation between the urine creatinine and the MRI, BIA and DXA measures of muscle mass being a good measure for estimating whole-body muscle mass [26, 31]. The CA-125, the folic acid and the urine creatinine shared the 86.6% variance in the non-con MT, adjusted by age, sex and BMI. Thus, these biomarkers could indicate MT loss in older adults with HFpEF.

The con MT showed a significant and moderate correlation with the folic acid (*r* = 0.532, *p* < 0.001) and a significant but poor correlation with the TSH (*r* = − 0.247, *p* = 0.037). The TSH was also related to sarcopenia in patients with HFrEF, so it could be another important biomarker to control and monitor changes in MT [54]. Moreover, the polypharmacy, the folic acid and other blood biomarkers explained the 38.5–44.4% variance of some US outcomes reported in our study, always adjusted by age, sex and BMI. Due to the complex pathophysiology of sarcopenia, it is unlikely that there will be a single biomarker that can identify sarcopenia [32, 33]. However, the present study results reported blood and urinary biomarkers that could be related to key pathophysiological processes in muscle mass loss in patients with HFpEF. These biomarkers were obtained by routine blood and urinary analyses and could monitor treatment effectiveness [27].

### Implications for clinical practice

Our results showed blood and urinary biomarkers related to pathophysiological mechanisms that lead to loss of muscle quantity and quality or to sarcopenia in patients with HFpEF. These biomarkers could allow monitoring of the effectiveness of treatment [27]. Exercise interventions can improve muscle strength, physical functional performance and muscle mass, so older adults with HFpEF should perform resistance exercise and aerobic exercise supervised by a physical therapist to improve these outcomes [55–58]. To improve muscle mass, older adults with HFpEF should perform extended resistance exercise programmes or higher doses and intensities of resistance exercise [56, 59, 60].

### Future research

Future research should assess the quadriceps femoris muscle activity by electromyography in older adults with HFpEF to assess nervous dysfunctions. Thus, an impaired electromyographic activity in older adults with HF has been reported, contributing to functional abnormalities of the skeletal muscle in the advanced stages of the HF [61]. More information should also be collected on HF and expected US abnormalities [62]. Future studies also should analyse US outcomes differences between older adults with HFpEF and older adults with HFrEF or healthy people. Future studies also could assess the effect of pharmacological and non-pharmacological treatments on these US outcomes. Kawai et al. [22] reported the possibility of using the US outcomes of the quadriceps femoris muscle to assess muscle strength, physical function, and sarcopenia in community-dwelling older adults. Thus, future studies should assess the relationship between US outcomes and muscle strength and physical functional performance in older adults with HFpEF. Moreover, future studies could confirm the findings shown by the present study, where the CA-125, the folic acid and the urine creatinine explained the 86.6% of the variance in the non-con MT, adjusted by age, sex and BMI. Future studies should also assess other biomarkers related to US outcomes.

### Strengths and limitations of the study

The main strength of our study is the description of the quantitative and qualitative characteristics of the quadriceps femoris, which had not been previously analysed in older adults with HFpEF. Moreover, the present study assessed the quantitative and qualitative characteristics of the quadriceps femoris in con and non-con situations. US assessment of con MT has shown to be an objective measure superior to the assessment of relaxed muscles [63]. Another strength of the study lies in the sample size. The author IJF-A conducted all the measurements to reduce the risk of bias among sonographers [64]. The US has also shown good intra-rater reliability [35] and excellent inter-rater reliability, regardless of the sonographer´s level of experience, the severity of patient illness, or patient setting [35, 65, 66]. Thus, the IJF-A experience should not have affected the results obtained on MT. All the US measurements were performed with the same US at the same point of the quadriceps femoris and with the same US parameters. The patients were also placed in the same chair and posture to avoid biases when obtaining the US outcomes. However, several limitations must be taken into account when interpreting the results. The landmark chosen to take the images and the patient´s position could affect the interpretation and comparison of the results across studies [67]. It was reported that the transducer should be placed at 1/2 or 2/3 the distance from the anterior superior iliac spine to the patella when quadriceps femoris MT want to be assessed [42, 65, 66, 68–71]. US measurements were taken in most previous studies with the patients in the supine position with their legs and knees extended and their muscles relaxed [35, 41, 42, 65, 66, 70]. We performed the US images with the patients seated with 90º of the knee and hip flexion, and US images were taken 15 cm from the upper edge of the patella. Thus, the US image landmark and the position of the patients may have affected the muscle thickness and the correlations shown in our study. Another limitation was the imputation of the acid folic, the CA-125, the urine creatinine and the AST data, which could have caused attrition bias in the results. The morphological characteristics of the included older adults could have affected the US outcomes, causing a detection bias in these outcomes. The statistically significant and the statistically non-significant results were presented in this study to avoid publication bias.

## Conclusion

Muscle wasting could reduce the aerobic capacity, the quality of life and the exercise intolerance of older adults with HFpEF. The US assessment could be the best tool to assess muscle quality and quantity in primary care settings. Moreover, the polypharmacy, blood and urinary biomarkers could be related to pathophysiological mechanisms of sarcopenia in older adults with HFpEF. However, future studies should confirm these findings.

## Supplementary Information

Below is the link to the electronic supplementary material.

Supplementary file1 (DOCX 33 KB)

Supplementary file2 (DOCX 19 KB)

## Data Availability

The data that support the findings of this study are available from the corresponding author, [AICV], upon reasonable request.

## References

[1] Bozkurt B, Coats AJ, Tsutsui H (2021). Universal definition and classification of heart failure: a report of the heart failure society of America, heart failure association of the European society of cardiology, Japanese heart failure society and writing committee of the universal definition o. J Card Fail.

[2] McDonagh TA, Metra M, Adamo M (2021). 2021 ESC Guidelines for the diagnosis and treatment of acute and chronic heart failure. Eur Heart J.

[3] Groenewegen A, Rutten FH, Mosterd A, Hoes AW (2020). Epidemiology of heart failure. Eur J Heart Fail.

[4] Jennifer E, Ho M, Danielle Enserro M (2016). Predicting heart failure with preserved and reduced ejection fraction: the international collaboration on heart failure subtypes. Circ Hear Fail.

[5] Dunlay SM, Roger VL, Redfield MM (2017). Epidemiology of heart failure with preserved ejection fraction. Nat Rev Cardiol.

[6] Ponikowski P, Voors AA, Anker SD (2016). 2016 ESC Guidelines for the diagnosis and treatment of acute and chronic heart failure. Eur Heart J.

[7] Anker SD, Coats AJS, Morley JE (2014). Muscle wasting disease: a proposal for a new disease classification. J Cachexia Sarcopenia Muscle.

[8] Bekfani T, Pellicori P, Morris DA (2016). Sarcopenia in patients with heart failure with preserved ejection fraction: Impact on muscle strength, exercise capacity and quality of life. Int J Cardiol.

[9] Curcio F, Testa G, Liguori I (2020). Sarcopenia and heart failure. Nutrients.

[10] Beltrami M, Milli M, Fumagalli C (2021). Frailty, sarcopenia and cachexia in heart failure patients: different clinical entities of the same painting. World J Cardiol.

[11] Konishi M, Kagiyama N, Kamiya K (2020). Impact of sarcopenia on prognosis in patients with heart failure with reduced and preserved ejection fraction. Eur J Prev Cardiol.

[12] Cruz-Jentoft AJ, Bahat G, Bauer J (2019). Sarcopenia: revised European consensus on definition and diagnosis. Age Ageing.

[13] Beaudart C, McCloskey E, Bruyère O (2016). Sarcopenia in daily practice: assessment and management. BMC Geriatr.

[14] Buckinx F, Landi F, Cesari M (2018). Pitfalls in the measurement of muscle mass: a need for a reference standard. J Cachexia Sarcopenia Muscle.

[15] Nijholt W, Scafoglieri A, Jager-Wittenaar H (2017). The reliability and validity of ultrasound to quantify muscles in older adults: a systematic review. J Cachexia Sarcopenia Muscle.

[16] Galindo Martín CA, Monares Zepeda E, Lescas Méndez OA (2017). Bedside ultrasound measurement of rectus femoris: a tutorial for the nutrition support clinician. J Nutr Metab.

[17] Perkisas S, Brockhattingen K, Welch C, Bahat G (2021). Using Ultrasound in the assessment of muscle. Eur J Geriatr Gerontol.

[18] Kim EY, Kim YS, Park I (2015). Prognostic significance of CT-determined sarcopenia in patients with small-cell lung cancer. J Thorac Oncol.

[19] Baracos VE, Reiman T, Mourtzakis M (2010). Body composition in patients with non-small cell lung cancer: a contemporary view of cancer cachexia with the use of computed tomography image analysis. Am J Clin Nutr.

[20] Akazawa N, Harada K, Okawa N (2018). Muscle mass and intramuscular fat of the quadriceps are related to muscle strength in non-ambulatory chronic stroke survivors: a cross-sectional study. PLoS ONE.

[21] Wilson DV, Moorey H, Stringer H (2019). Bilateral anterior thigh thickness: a new diagnostic tool for the identification of low muscle mass?. J Am Med Dir Assoc.

[22] Kawai H, Kera T, Hirayama R (2018). Morphological and qualitative characteristics of the quadriceps muscle of community-dwelling older adults based on ultrasound imaging: classification using latent class analysis. Aging Clin Exp Res.

[23] Landi F, Calvani R, Lorenzi M (2016). Serum levels of C-terminal agrin fragment (CAF) are associated with sarcopenia in older multimorbid community-dwellers: Results from the ilSIRENTE study. Exp Gerontol.

[24] Chew J, Tay L, Lim JP (2019). Serum Myostatin and IGF-1 as gender-specific biomarkers of frailty and low muscle mass in community-dwelling older adults. J Nutr Heal Aging.

[25] Chung TH, Kwon YJ, Shim JY (2016). Association between serum triglyceride to high-density lipoprotein cholesterol ratio and sarcopenia in elderly Korean males: the Korean national health and nutrition examination survey. Clin Chim Acta.

[26] Clark RV, Walker AC, Miller RR (2018). Creatine (methyl-d3) dilution in urine for estimation of total body skeletal muscle mass: accuracy and variability vs MRI and DXA. J Appl Physiol.

[27] Calvani R, Picca A, Marini F (2020). Identification of biomarkers for physical frailty and sarcopenia through a new multi-marker approach: results from the BIOSPHERE study. GeroScience.

[28] Kim SH, Kwon HS, Hwang HJ (2017). White blood cell counts, insulin resistance, vitamin D levels and sarcopenia in Korean elderly men. Scand J Clin Lab Invest.

[29] Tanaka M, Okada H, Hashimoto Y (2020). Association of mean corpuscular volume with sarcopenia and visceral obesity in individuals without anemia. J Diabetes Investig.

[30] Wang N, Chen M, Fang D (2020). Relationship between serum triglyceride to high-density lipoprotein cholesterol ratio and sarcopenia occurrence rate in community-dwelling Chinese adults. Lipids Health Dis.

[31] Buehring B, Siglinsky E, Krueger D (2018). Comparison of muscle/lean mass measurement methods: correlation with functional and biochemical testing. Osteoporos Int.

[32] Curcio F, Ferro G, Basile C (2016). Biomarkers in sarcopenia: a multifactorial approach. Exp Gerontol.

[33] Tosato M, Marzetti E, Cesari M (2017). Measurement of muscle mass in sarcopenia: from imaging to biochemical markers. Aging Clin Exp Res.

[34] Lopez P, Pinto MD, Pinto RS (2019). Does rest time before ultrasonography imaging affect quadriceps femoris muscle thickness, cross-sectional area and echo intensity measurements?. Ultrasound Med Biol.

[35] Tourel C, Burnol L, Lanoiselé J et al (2020) Reliability of standardized ultrasound measurement of quadriceps muscle thickness in critically ill neurological patients: comparison with computed tomography measures. J Rehabil Med. 10.2340/16501977-2638 10.2340/16501977-2638 31922203

[36] Ishida Y, Carroll JF, Pollock ML (1992). Reliability of B-mode ultrasound for the measurement of body fat and muscle thickness. Am J Hum Biol.

[37] Association WM (2013). World medical association declaration of Helsinki: ethical principles for medical research involving human subjects. JAMA.

[38] Vandenbroucke JP, von Elm E, Altman DG, Gotzsche PC, Mulrow CD, Pocock SJ, Poole C, Schlesselman JJEM (2007). Strengthening the reporting of observational studies in epidemiology (STROBE): explanation and elaboration. Ann Intern Med.

[39] von Elm E, Altman DG, Egger M, Pocock SJ, Gotzsche PCVJ (2007). The strengthening the reporting of observational studies in epidemiology (STROBE) statement: guidelines for reporting observational studies. Lancet.

[40] Watanabe Y, Yamada Y, Fukumoto Y (2013). Echo intensity obtained from ultrasonography images reflecting muscle strength in elderly men. Clin Interv Aging.

[41] Morimoto Y, Kawano H, Miyanaga K (2020). Association of lower extremity function with nutritional status and number of drugs in patients with chronic heart failure. J Int Med Res.

[42] Nakano I, Hori H, Fukushima A (2020). Enhanced echo intensity of skeletal muscle is associated with exercise intolerance in patients with heart failure. J Card Fail.

[43] Lena A, Anker MS, Springer J (2020). Muscle wasting and sarcopenia in heart failure—the current state of science. Int J Mol Sci.

[44] Tucker WJ, Haykowsky MJ, Seo Y (2018). Impaired exercise tolerance in heart failure: role of skeletal muscle morphology and function. Curr Heart Fail Rep.

[45] Kinugasa Y, Yamamoto K (2017). The challenge of frailty and sarcopenia in heart failure with preserved ejection fraction. Heart.

[46] Bekfani T, Bekhite Elsaied M, Derlien S (2020). Skeletal muscle function, structure, and metabolism in patients with heart failure with reduced ejection fraction and heart failure with preserved ejection fraction. Circ Hear Fail.

[47] König M, Spira D, Demuth I (2017). Polypharmacy as a risk factor for clinically relevant sarcopenia: results from the berlin aging study II. J Gerontol A Biol Sci Med Sci.

[48] Agosta L, Bo M, Bianchi L (2019). Polypharmacy and sarcopenia in hospitalized older patients: results of the GLISTEN study. Aging Clin Exp Res.

[49] Hung CL, Hung TC, Liu CC (2012). Relation of carbohydrate antigen-125 to left atrial remodeling and its prognostic usefulness in patients with heart failure and preserved left ventricular ejection fraction in women. Am J Cardiol.

[50] Llàcer P, Bayés-Genís A, Núñez J (2019). Carbohydrate antigen 125 in heart failure a new era in the monitoring and control of treatment. Med Clínica English Ed.

[51] Cruz-Jentoft AJ, Sayer AA (2019). Sarcopenia. Lancet.

[52] Mithal A, Bonjour JP, Boonen S (2013). Impact of nutrition on muscle mass, strength, and performance in older adults. Osteoporos Int.

[53] Seino S, Sumi K, Narita M (2018). Effects of low-dose dairy protein plus micronutrient supplementation during resistance exercise on muscle mass and physical performance in older adults: a randomized, controlled trial. J Nutr Heal Aging.

[54] Canteri AL, Gusmon LB, Zanini AC (2019). Sarcopenia in heart failure with reduced ejection fraction. Am J Cardiovasc Dis.

[55] Escriche-Escuder A, Fuentes-Abolafio IJ, Roldán-Jiménez C (2021). Effects of exercise on muscle mass, strength, and physical performance in older adults with sarcopenia: a systematic review and meta-analysis according to the EWGSOP criteria. Exp Gerontol.

[56] Beckwée D, Delaere A, Aelbrecht S, Gerontology Dgotbso, (BSGG) AG (2019). Exercise interventions for the prevention and treatment of sarcopenia a systematic umbrella review. J Nutr Heal Aging.

[57] Bao W, Sun Y, Zhang T (2020). Exercise programs for muscle mass, muscle strength and physical performance in older adults with sarcopenia: a systematic review and meta-analysis. Aging Dis.

[58] Vlietstra L, Wendy H (2018). Exercise interventions in healthy older adults with sarcopenia: a systematic review and meta-analysis. Australas J Ageing.

[59] Fuentes-Abolafio IJ, Stubbs B, Pérez-Belmonte LM (2020). Physical functional performance and prognosis in patients with heart failure: a systematic review and meta-analysis. BMC Cardiovasc Disord.

[60] Francaux M, Deldicque L (2019). Exercise and the control of muscle mass in human. Pflugers Arch Eur J Physiol.

[61] Schulze PC, Linke A, Schoene N (2004). Functional and morphological skeletal muscle abnormalities correlate with reduced electromyographic activity in chronic heart failure. Eur J Prev Cardiol.

[62] Wijntjes J, van Alfen N (2021). Muscle ultrasound: present state and future opportunities. Muscle Nerve.

[63] Abraham A, Fainmesser Y, Lovblom LE (2020). Superiority of sonographic evaluation of contracted versus relaxed muscle thickness in motor neuron diseases. Clin Neurophysiol.

[64] Segers J, Hermans G, Charususin N (2015). Assessment of quadriceps muscle mass with ultrasound in critically ill patients: intra- and inter-observer agreement and sensitivity. Intensive Care Med.

[65] Mayer KP, Dhar S, Cassity E (2020). Interrater reliability of muscle ultrasonography image acquisition by physical therapists in patients who have or who survived critical illness. Phys Ther.

[66] Vieira L, Rocha LPB, Mathur S (2019). Reliability of skeletal muscle ultrasound in critically ill trauma patients. Rev Bras Ter Intensiva.

[67] Perkisas S, Baudry S, Bauer J et al (2018) Application of ultrasound for muscle assessment in sarcopenia: towards standardized measurements. Eur Geriatr Med 9(6):739–757. 10.1007/s41999-018-0104-9 10.1007/s41999-018-0104-9 34674473

[68] Bickerstaffe A, Beelen A, Zwarts MJ (2015). Quantitative muscle ultrasound and quadriceps strength in patients with post-polio syndrome. Muscle Nerve.

[69] Martínez-Payá JJ, del Baño-Aledo ME, Ríos-Díaz J (2017). Muscular echovariation: a new biomarker in amyotrophic lateral sclerosis. Ultrasound Med Biol.

[70] Cruz-Montecinos C, Guajardo-Rojas C, Montt E (2016). Sonographic measurement of the quadriceps muscle in patients with chronic obstructive pulmonary disease: functional and clinical implications. J Ultrasound Med.

[71] Nijboer-Oosterveld J, Nens VA, Pillen S (2011). New normal values for quantitative muscle ultrasound: obesity increases muscle echo intensity. Muscle Nerve.

