# Supplementary material for: Biomarkers and the quadriceps femoris muscle architecture assessed by ultrasound in older adults with heart failure with preserved ejection fraction: a cross-sectional study
Source: Aging Clin Exp Res. 2022 Aug 8;34(10):2493–504. doi: 10.1007/s40520-022-02189-7 (PMC9637604; doi:10.1007/s40520-022-02189-7)
Supplement: Supplementary file 2 — Supplementary file2 (DOCX 19 KB) [file 40520_2022_2189_MOESM2_ESM.docx]

| **Appendix B.** Participant descriptive, anthropometric and clinical variables (n=76). | | | | | | | |  |  |
| --- | --- | --- | --- | --- | --- | --- | --- | --- | --- |
|  | | **Mean (SD)** | | | **Min-Max** | | |  |  |
| Age (years) | | 80.75 (5.90) | | | 70.0-96.0 | | |  |  |
| LVEF (%) | | 60.74 (4.43) | | | 50.0-75.0 | | |  |  |
| Comorbidities (n) | | 8.41 (1.91) | | | 4.0-14.0 | | |  |  |
| Height (m) | | 1.61 (0.08) | | | 1.43-1.84 | | |  |  |
| Weight (kg) | | 76.73 (14.97) | | | 52.0-116.50 | | |  |  |
| BMI (kg/m^2^) | | 29.69 (5.86) | | | 19.81-51.78 | | |  |  |
| Polypharmacy (n) | | 10.18 (3.15) | | | 3.0-19.0 | | |  |  |
|  | **n (Percentage)** | | |  | | | |  |  |
| **Drugs** | | |  | | | |  | | |
| ACE inhibitors  ARB  Beta-Blockers  Ivabradine  Ca-antagonists  Loop Diuretics  Mineralocorticoid Receptor Antagonist  Thiazide  Nitrates  Acarboxyprothrombin  Factor Xa Inhibitors  Acetylsalicylic acid  Hypoglycemic Agents  Metformin  SGT2I  GLP1  DPP4  Insulin | | | 16 (21.10%)  47 (61.80%)  56 (73.70%)  4 (5.30%)  22 (28.90%)  65 (85.50%)  24 (31.60%)  10 (13.20%)  21 (27.60%)  14 (18.40%)  31 (40.80%)  26 (34.20%)  37 (48.70%)  18 (23.70%)  9 (11.80%)  2 (2.60%)  20 (26.30%)  21 (27.60%) | | |  | | |  |
| **Comorbidities** | | |  | | |  | | |  |
| Arterial Hypertension | | | 74 (97.40%) | | |  | | |  |
| DM | | | 44 (57.90%) | | |  | | |  |
| Dyslipidemias | | | 66 (86.80%) | | |  | | |  |
| [Atrial Fibrillation](https://www.ncbi.nlm.nih.gov/mesh/68001281) | | | 44 (57.90%) | | |  | | |  |
| COPD | | | 21 (27.60%) | | |  | | |  |
| Stroke | | | 10 (13.20%) | | |  | | |  |
| CRI | | | 49 (64.50%) | | |  | | |  |
| OSA | | | 16 (21.10%) | | |  | | |  |
| Cognitive Impairment | | | 8 (10.50%) | | |  | | |  |
| Anemia | | | 36 (47.40%) | | |  | | |  |
| PAD | | | 4 (5.30%) | | |  | | |  |
| Depression | | | 26 (34.20%) | | |  | | |  |
| Cancer Disease | | | 12 (15.80%) | | |  | | |  |
| LVH | | | 41 (53.90%) | | |  | | |  |
| LVD | | | 72 (94.70%) | | |  | | |  |
| LAD | | | 37 (48.70%) | | |  | | |  |
| Heart Valve Disease | | | 50 (65.80%) | | |  | | |  |
| Aortic Valve Disease | | | 25 (32.90%) | | |  | | |  |
| Mitral Valve Disease | | | 35 (46.10%) | | |  | | |  |
| Tricuspid Valve Disease | | | 17 (22.40%) | | |  | | |  |
| Pulmonary Hypertension | | | 13 (17.10%) | | |  | | |  |
| **NYHA**  *II*  *III* | | | 53 (69.70%)  25 (30.30%) | | |  | | |  |
| **BMI**  *Normal Weight*  *Overweight*  *Obesity* | | | 16 (21.1%)  31 (40.8%)  29 (38.2%) | | |  | | |  |
| **Gender**  female  male | | | 45 (59.20%)  31 (40.80%) | | |  | | |  |
| **History of Smoking**  *No*  *Yes* | | | 50 (65.80%)  26 (34.20%) | | |  | | |  |
| **Number of falls in the last year**  *0*  *1*  *2*  *3*  *> 3* | | | 34 (44.70%)  20 (26.30%)  9 (11.80%)  7 (9.20%)  6 (7.90%) | | |  | | |  |
| **Marital Status**  *Single*  *Married*  *Divorced*  *Widower* | | | 6 (7.90%)  32 (42.10%)  1 (1.30%)  37 (48.70%) | | |  | | |  |
| **Academic Degree/Studies Level**  *Not knowing how to read or write*  *Literacy*  *Primary studies*  *Secondary studies*  *Higher education* | | | 11 (14.50%)  35 (46.10%)  13 (17.10%)  6 (7.90%)  11 (14.50%) | | |  | | |  |

**SD**: Standard Deviation; **LVEF:** Left Ventricular Ejection Fraction; **BMI:** Body Mass Index; **ACE inhibitors:** Angiotensin Converting Enzyme Inhibitors; **ARB:** Angiotensin II Receptor Blocker; **SGT2I:** Sodium-Glucose Transporter 2 Inhibitors; **GLP1:** Glucagon-Like Peptide 1; **DPP4:** DiPeptidyl Peptidase-4; **DM**: Diabetes Mellitus; **COPD**: Chronic Obstructive Pulmonary Disease; **CRI**: Chronic Renal Insufficiency; **OSA**: Obstructive Sleep Apnea; **PAD**: Peripheral Arterial Disease; **LVH**: Left Ventricular Hypertrophy; **LVD**: Left Ventricular Dilatation; **LAD**: Left Atrial Dilatation; **NYHA:** New York Heart Association class.
